# Using Solution-Focused Coaching in Social Work Practice with the Long-Term Unemployed to Promote Their Well-Being

**DOI:** 10.3390/ijerph20065180

**Published:** 2023-03-15

**Authors:** Leoš Zatloukal, Tatiana Matulayová, Pavlína Jurníčková, Nataša Matulayová, Jakub Doležel, Hana Šlechtová

**Affiliations:** Department of Christian Social Work, Sts Cyril and Methodius Faculty, Palacký University Olomouc, 779 00 Olomouc, Czech Republic

**Keywords:** social work, coaching, unemployment, solution-focused approach, Reteaming

## Abstract

Long-term unemployment, one of the challenges social workers face, produces a variety of non-monetary and social consequences. The helping professionals understand that interventions towards unemployed clients do not cover just the pure fact of their unemployment but that a holistic approach to the clients’ living situations is needed. This paper aims to promote well-being while implementing solution-focused coaching in working with unemployed clients in social work practice. The Reteaming coaching model is supported by two detailed case studies describing three key areas covered in the Reteaming process. Working with clients in both cases contributed to various elements of the client’s psychological well-being: positive emotions, engagement, relationships, meaning, and achievement. The Reteaming coaching model can be used effectively as a suitable structured approach, mainly used in strength-based social work.

## 1. Introduction

Long-term unemployment represents a serious problem with many psychological, economic, and social consequences [1,2,3]. Employment is an important support in the construction of social identity and of self-identity as such. Success in the labor market becomes a support for self-esteem. On the contrary, a more permanent identification with the position of the unemployed may contribute to a decrease in self-esteem, and thus to a deterioration of both subjective well-being (a bad feeling about oneself and about one’s performance) and its “objective” dimensions (through worsening of one’s income situation or a reduction in the network of social contacts). Thus, naturally, long-term unemployment constitutes a significant part of the concern of social workers [4].

This article focuses on the well-being of unemployed clients and the possibilities of solution-focused coaching used with those clients to identify their resources and/or “create”/“construct” them (through coaching interaction) and enhance the clients’ well-being both during the search and through finding an employment. The article aims to promote well-being while implementing solution-focused coaching in working with unemployed clients in social work practice.

The first part of the text deals with well-being (particularly psychological well-being) and its relationship with work and unemployment. The second part addresses the context of the presented work—the use of coaching in social work practice. As Caspi points out, coaching represents many possibilities and challenges for the social work profession [5]. Since discussion of the topic is relatively scarce, we try to contribute briefly to this discussion based on our practice. Solution-focused coaching, particularly the Reteaming model, is introduced in the last part of the article. Three main areas of solution-focused coaching—negotiating direction, resources, and action—are described, and some basic methods are presented there too. Two detailed case examples are available to illustrate the application of the Reteaming model. Although the effectiveness of solution-focused coaching while working with unemployed clients is still poorly established by sound research studies, we can link the coaching methods used and the promotion of clients’ psychological well-being. 

## 2. Psychological Well-Being and Long-Term Unemployment

Well-being is a concept that generally refers to whatever is assessed in evaluating a person’s life situation or being, so it describes the state of one’s life situation [6]. This concept is thus very broad and many other partially overlapping terms, such as “happiness”, “quality of life”, “welfare”, “well-living”, “life satisfaction”, “prosperity”, or “empowerment” point in the same direction [6,7,8,9]. Well-being is a multidimensional concept [10] and can be viewed as an adaptive process from the perspective of the individual, organization(s), and community [11]. Veenhoven distinguishes between “inner” and “other” well-being (focus on individual or environment/context) and between “input” and “outcome” well-being in [8]. Well-being is sometimes divided into domains such as psychological (subjective) well-being [9], social well-being [12], and spiritual well-being [13], among others. Psychological well-being is, according to Diener [10], 1. subjective (resides within the experience—cognitive as well as affective—of the individual), 2. positive (not only the absence of negative factors), and 3. global (not focused only to one narrow dimension sense).

Although this text focuses on subjective and psychological well-being [7,9,10], we need to understand this concept in its general social context. Here, the sociological theory of identity developed by Kaufmann [14] may be helpful. Kaufmann defines identity as an image, idea, or sensation of the self and at the same time as an uninterrupted life-long process, which in every moment enables an individual to perceive him-/herself as total, as all the time him-/herself. In our contemporary society, Kaufmann [14] writes that identity enables individuals to act, as action does no longer derive clearly from the social role. Except for repetitive, routine, unreflected behavior, identity is a condition of action, and one cannot act without being able to imagine him-/herself acting like that. Thus, an individual’s identity is always a certain distance to his/her present state. However, for an identity (and subsequent action) to be truly possible, the idea cannot remain at the level of dreaming; its realization must be made possible by the individual’s resources: economic, social, cultural, educational, and other.

Another trait of contemporary social context is pressure towards relentless creativity in the sphere of identity: an individual is pushed to imagine and invent him-/herself differently (even without the individual’s own desire). Failure to meet this requirement compromises the individual’s self-esteem, which can also result in a socially induced depressive state, i.e., an obvious deterioration in subjective well-being. To avoid it, individuals tend to react in three ways: externalized outbursts of emotion (voice), withdrawal and clinging to routine and modest material safety (exit), or an even stronger clinging to a role that provides them with abundant resources, but at the same time entails the impossibility of that very identity creativity (loyalty), depending on their openness/closedness to such a process and on the resources potentially enabling the translation of the imagined identity creativity into real action. It is equally important, however, that the individual in question be aware of his or her sources in some way or even draw on them unreflectively. However, a long-term unemployed person may tend to underestimate or ignore his or her resources. A coach can be helpful as an actor with whom the individual interacts to reshape his/her identity (which, as mentioned, is an ongoing, lifelong process) in a direction that is desirable for finding employment or employment in the labor market. 

Well-being is not only a multidimensional concept but also a processual one. Even if it is referred to and measured as a state [10], it can be better understood as a complex dynamic adaptive process [11,15,16]. Some authors document this by presenting not only lists of variables or factors but rather some dynamic “trajectories” such as upward and downward “spirals” [7,17] or a “human development path” [8].

There are some basic elements of psychological well-being described in the literature, incl. well-known Ryff and Seligman typologies. Ryff describes six dimensions of well-being: 1. self-acceptance, 2. positive relations with others, 3. autonomy, 4. environmental mastery, 5. purpose in life, and 6. personal growth [18,19]. Seligman refers to five well-being elements summed up in PERMA: 1. positive emotions, 2. engagement (flow), 3. relationships, 4. meaning, and 5. achievement [1]. Those lists of elements serve as a map for orientation in promoting well-being endeavors.

Work is an important domain of psychological well-being. The relationship between work and psychological well-being is relatively complex [20]. Work enhances psychological well-being if it is perceived as satisfying and secure [21], but at the same time, it enhances stress levels [20,21]. A similar complex relationship was found between unemployment and psychological well-being [20,22]. The results of unemployment include the loss of income together with a lower standard of living, but also changes in social status, isolation of unemployed people, development of negative self-assessment, changes in self-identity, family disturbance, community relationships, or somatic problems [3,22,23,24,25]. On the other hand, most of the impacts of unemployment have been found in the cognitive domain of well-being (such as status) and they have much less impact on affective well-being and eudaimonic (personal growth and a sense of purpose) well-being [22]. Some studies have shown the negative impact of unemployment on mental health, such as hopelessness, lowered self-esteem, social isolation, resigned apathy, or disintegration, while in some minority cases, the opposite (gain of mental health) was found [20]. Some studies have also documented the influence of socioeconomic situations on psychological well-being [18]. Ryff’s study show that a lower socioeconomic level leads to lower self-acceptance, purpose in life, and personal growth [18]. Diener showed that the change in a person’s income had a bigger impact on well-being than the absolute levels of income themselves [10,18]. This emphasizes the issue of adaptation in the situation of unemployed people [22]. We can summarize that the impacts of unemployment on psychological well-being differ due to the reason of work lost, age, personality, gender, level of satisfaction or stress in previous work, social support, length of unemployment, and many other factors, and those impacts of unemployment on psychological well-being are mostly (but not only) negative [18,20,21,22,26]. Unemployed people often seek help from social workers and other professionals. Social workers use various methods to help people [27,28]. The next section of the text focuses on social work practice and using coaching to work with unemployed people.

## 3. Application of Coaching in Social Work Practice 

Long-term unemployment represents a serious problem with a mostly negative impact on a person’s well-being, and naturally, it constitutes a very significant part of the concern of social workers, especially those specialized in counseling [4,29,30]. Although a solution-focused approach is well established in social work practice [27,28,31,32,33,34], using coaching (and particularly solution-focused coaching) in social work practice is still an open question with only a few attempts to answer it [5,35,36,37]. 

First, there are many various definitions of coaching. Whittmore, in his classic book, writes: “Coaching is unlocking people’s potential to maximize their performance. It is helping them to learn rather than teaching them.” [38] (p. 10). Downey proposed a similar definition: “Coaching is about the art of facilitating the performance, learning, and development of others” in [39] (p. 1) Another definition by Grant and Palmer emphasizes “enhancing well-being and performance in personal life and work domains, underpinned by models of coaching grounded in established learning theories and psychological approaches” in [40] (p. 8).

The definitions above are rather general, but they all emphasize growth as a goal (e.g., performance, learning, and well-being) and link coaching closely to learning. At the same time, the learning process is perceived in coaching in a very specific way [38,39,41,42]. While “traditional learning” stresses the importance of the trainer and his/her role in determining learning goals, showing the “right” technique, correcting mistakes, and taking responsibility for the learning process as a whole, coaching emphasizes goals defined by the client, searching for the best fit between techniques and the client’s unique resources, focusing on the client’s strengths and the shared responsibility of the client and coach [40,41,42]. Metaphorically, traditional learning is like filling the glass up while coaching associates an acorn that needs the appropriate conditions for its growth [43].

Methods and techniques, applied in social work interventions, often come from different environments, e.g., coaching has evolved in business. Nevertheless, can it be applied in social work and counseling practice? If so, can it truly bring something new because there are various approaches and models within social work practice? Our answer to both questions is a cautious “yes”. First, coaching corresponds appropriately with current trends in social work, such as the “participation” of clients and individual approaches to clients [5,28]. It also fits to “managerial” context of social work where (perhaps paradoxically) coaching can open new possibilities for relationship-based social work [36]. Secondly, coaching offers a non-pathology frame of work with clients because coaching is, by definition, focused on growth and does not require any identified “pathology” to operate itself [5,35]. From this point of view, coaching is very close to social work practice [5,44], especially to strength-based and solution-focused approaches well established in social work practice [27,30,44,45,46,47]. Furthermore, we can see possibilities for using coaching not only in the counseling practice of social workers itself but also for the structural support of stakeholders (e.g., in making positive changes in the labor market) and for the support of social workers as well [30,48,49,50].

What leads to caution is mainly the imprecise definition of coaching as an activity or profession, as well as a lack of clear standards and authorities which would guarantee that not everyone can claim to be a coach even without the relevant qualification requirements and adherence to ethical standards of the profession [5,51].

There seems to be an appropriate space for integrating coaching, especially in social work. As Caspi commented, “Social work is perhaps the best positioned to take the leadership role in coaching, and social workers are well-suited to become coaches… they have expertise in helping individuals overcome personal and interpersonal barriers of growth.” [5] (p. 260). The simplest way is to let social workers qualify as coaches, which is already the case in some countries [35]. On the other hand, counselors (including social workers) have been “relatively slow in positioning themselves in the coaching market” [51] (p. 347). This has left others, possibly less qualified, to perform the job and focus on goal attainment, self-discovery, and personal development issues [51]. We can speculate about the reasons (e.g., training in coaching is relatively expensive, and there are no clear standards for coaching as a profession), but at the same time, we can think of coaching as a new possibility for social workers and their professional development [5]. As one study shows, many social workers already use coaching methods in their practice, even if they do not define themselves as coaches [35]. 

The promising area of integrating coaching into social work practice lies in professional counseling work with unemployed clients. For coaching in social work with unemployed clients, we can simply use the broad terms “career coaching” or “job coaching” [25,52,53,54]. Both terms suggest a focus on growth—in career development or job change and/or preservation—and do not invite an impoverishing frame (e.g., dealing with “underlying problems”, “solving unemployment”, “doing treatment”, and “advising by an expert”). Considering the importance of the self-definition of the professional [55] and the importance of naming things in the construction of social reality [56], this distinction is very important. The following parts introduce the coaching model Reteaming based on the solution-focused approach and its use in a social worker’s career/job coaching consultations with unemployed clients.

## 4. Solution-Focused Coaching with Unemployed Clients

Solution-focused coaching evolved from solution-focused therapy as an application of its principles and techniques in a new context [43,57,58,59]. Solution-focused therapy evolved in the 1980s in the Brief Family Therapy Centre Milwaukee (USA) as a goal-oriented and resource-oriented approach [60,61], which is similar to coaching focus [57]. Solution-focused brief therapy (SFBT) is one of the systems of psychotherapy rooted in family therapy [61]. Its roots can be traced back to the work of the MRI Institute in Palo Alto and to the late Milton H. Erickson [60], and it evolved out of the clinical practice of Steve de Shazer, Insoo Kim Berg, and colleagues at the Brief Family Therapy Center in Milwaukee, Wisconsin, in the early 1980s [61,62]. SFBT is grounded in postmodern philosophy (constructivism and social constructionism) and in late Wittgensteinian philosophy [58,61,62] and became well-known in the field of psychotherapy for the pragmatic focus on solution-development (instead of analyzing problems and trying to fix them), preferred future and resource orientation, and ongoing research (particularly recording and analyzing therapeutic sessions and collecting data about the outcome of therapy from clients) [32,61,63]. Relatively soon was this therapeutic approach adapted to other professional fields such as supervision, mediation, and coaching [58,62,63]. We would like to introduce the solution-focused coaching model Reteaming, used in working with unemployed clients.

The Reteaming model was created by Ben Furman and Tapani Ahola from Finland [64]. It was originally designed as a model for coaching teams and groups and later adopted for coaching individuals. Reteaming has been defined by the authors themselves as follows: “Reteaming is a generic, multipurpose method consisting of 12 steps intended to help individuals as well as groups of people to change for the better by facilitating the setting of goals and increasing motivation and enhancing cooperation needed to achieve them.” [64] (p. 9) The twelve steps mentioned here are [64]: 1. describe your dream (vision of preferred future), 2. identify a goal, 3. recruit supporters, 4. highlight the benefits of the goal, 5. recognize progress already made, 6. picture forthcoming progress, 7. acknowledge the challenge, 8. find grounds for confidence, 9. make your promise, 10. follow up on your progress, 11. prepare for possible setbacks, and 12. celebrate success and acknowledge your supporters. These steps are not understood as a strict, unchangeable “manual” but rather as a guide for creative and individualized work with people. Reteaming offers a basic structure for effective interviews that enables creative improvisation while helping novice coaches learn the basic skills of the solution-focused approach and keep the key tenets of this approach [62,64]. 

Three main areas were mentioned in the definition of Reteaming—goals, motivation, and cooperation. Starting with the last one, cooperation plays a crucial role in a solution-focused approach [31,63,65]. In Reteaming, coaches do not work with the assumption that (at least some) clients are “non-cooperative”, “resistant”, or “unmotivated” but assume that clients are always prepared and willing to cooperate even though in their unique way (which is not always the way a worker would prefer) [31,65,66,67]. From this point of view, “working alliance” is always of more importance than any technique in coaching [41,68]. Working alliance is a well-established concept in counseling and psychotherapy research that stresses the importance of emotional bond (the client feels listened to, safe, and acknowledged), agreement on tasks (methods used by the practitioner), and agreement on goals [41,68,69]. This links to a second area in Reteaming’s definition—goals. Goals are central in Reteaming because they build a frame for cooperation and simultaneously foster motivation for a desired change. Furman and Ahola present five factors that enhance motivation [64], which correspond with research findings from different fields:

1. The goal of the client—if clients choose the goal by themselves, their motivation is going to be higher than if they are pushed toward something [70].
2. The goal has a value for the client—if the goal is valuable for the client, the client feels some “passion” for it, and its achievement will bring some benefit, then there is also higher motivation to work at it [70,71,72]. A valuable goal contributes to the felt meaning of life and the client’s well-being [72,73,74,75].
3. The goal is achievable—an unrealistic goal reduces motivation; a goal that the client believes is achievable increases motivation [71,76,77,78,79]. Many studies have also shown the importance of concepts such as the sense of coherence or self-efficacy for psychological well-being [80,81,82].
4. There is some (however small) progress towards the goal—only a few people can keep doing something with enthusiasm when it either fails or shows no progress; goal theory emphasizes contingent and non-contingent rewards [83].
5. The client is prepared to deal with possible setbacks—the emergence of setbacks can significantly reduce motivation, especially when someone is not prepared for them, whereas anticipated obstacles can strengthen the commitment of the client to overcome them [78]. Many studies have documented the importance of coping strategies [84], which are considered to be mediators between global and situational meanings (e.g., goals, beliefs, and subjective feeling of meaning in life) and psychological well-being in some cases [85].

It is worth noting that the 12 previously mentioned steps of Reteaming somehow cover these five motivation factors. In practical terms, we can describe three key areas to which we pay close attention in Reteaming: they can be called “negotiating direction” (connected especially with steps 1, 2, and 4), “resources” (connected especially with steps 5, 3, 8, and 10), and “action” (connected especially with steps 6, 9, and 11). These individual areas are covered in the following part of the article, and they are illustrated with the help of practical examples.

### 4.1. Negotiating the Direction

In Reteaming (and solution-focused approaches in general), much attention is paid to the thick description of the client’s vision of their preferred future [57,58]. The conversation about the vision of the preferred future is generally based on the “miracle question” or its alternatives [58]. From the description of a preferred future in Reteaming, we usually move on to the goal, which is necessary to achieve to realize the dream/preferred future [64]. The goal is more specific than the preferred future. Frequently, there are several goals from which clients choose one to begin with. It proves useful to name the goal or even to depict it symbolically. This process allows clients to work on the definition of goals creatively in terms of metaphors [86], and consequently, it enriches the definition of the goal itself. Some clients put their symbols as a screensaver on their laptops or wallpaper on their mobile phones as a reminder to themselves. It is also possible not to clarify goals but to work on the vision of the preferred future or small signs of improvement instead [39,58].

A key aspect is finding the benefits of the goals for the client and the other people involved. One of the motivating factors mentioned above is that the goal has value for the client. The very discussion about the benefits of the goal strengthens the awareness of these benefits and thereby enhances the client’s motivation [64].

#### 4.1.1. Case Example 1: Mrs. A

Both cases A and B mentioned in this text took place in cooperation with the employment office, which selected several long-term unemployed clients or clients at risk of long-term unemployment for individual coaching sessions with one of the authors (LZ) and other coaches. All of the clients were asked if they wanted to participate in this coaching project. Those who agreed had the opportunity to have a maximum of ten coaching sessions for free. Scheduling dates of meetings was up to clients and coaches, the only rule was that they have to fit in one year-long project period. Informed consent was obtained from both clients mentioned in this text; names and significant details were changed to protect the confidentiality of the clients. The presented two case examples were selected to illustrate different and flexible ways of using the Reteaming model with unemployed clients.

I (L.Z.) had a session with a 46-year-old woman who looked after her 7-year-old son and was recommended for a consultation by the employment office. Before her maternity leave, she had worked at one of the biggest Czech banks as the head of the market analysis department for 17 years. When she was supposed to go back to work after her maternity leave, she was not interested in getting her old job back as she would have had to devote such an enormous amount of time to her work that she would not have the chance to also look after her son, and she did not want to have a “workaholic” lifestyle. The client kept speaking about the details and reasons that prevented her from finding a job. I kept listening carefully and complimented her on her many attempts to resolve her situation. Sometime later, I encouraged her to describe her preferred future: “If you had a perfect life and you could have the job you wanted, what would your life be like?”

*“I would work well in a team. It would be a kind of calm job and specialized in some way… financial gain is not my priority anymore. The most important thing is looking after my son. I used to have a very well-paid job, but it was time-consuming, and I would not like things to be like that now.”*

When we moved on in her description and spoke more specifically, she mentioned she would like to work in the economic sector, ideally having a part-time job (although it was not a necessary condition). The optimum employer would be the city council, district office, or an institution such as an NGO. The minimum income she needs is CZK 13,000 (this was much less than the average income in the Czech Republic and even much less than in her previous job), and she would receive other benefits instead (e.g., being in contact with people, self-fulfillment, and having enough time to be with her son). During the session, we spoke about her vision and the complaints and obstacles that the client tended to emphasize. I listened carefully and confirmed my understanding of her worries, but simultaneously made her go back to the topic of her preferred future and describe it in greater detail. Speaking about her vision and its benefits for her and her son made her very enthusiastic, and her eyes shone with enthusiasm.

#### 4.1.2. Case Example 2: Mr. B

A 33-year-old male postgraduate of biology was recommended for a consultation with me by the Employment Office, and his work experience included a year at the post office (sorting packages) and intermittent fieldwork as a biologist. From the very beginning, he expressed great skepticism about his ability to succeed in obtaining a job. He described himself as a solitary person who is not very communicative. When asked about his dream, he responded in negative terms and shared what he does not want: to work in a place where they require fluent English; to work night shifts; to work for a company that uses only telephone contact (he is willing to respond to offers only in written form, i.e., e-mails); to work if he is supposed to drive a car; to work outside the city where he lives; and to work if he needs a trade certificate. 

This list has limited possibilities for the client because very few job positions could meet the above standards and are not already occupied. Later on, though very slowly, we managed to discuss his preferences and wishes. He talked about his wish to get a staid job, preferably out in the field (e.g., biology, nature conservation, or at a zoo) or in administration. The salary is not a determining factor; however, he would like to earn at least CZK 10,000 per month. When we spoke about the benefits of such a job, he seemed interested and passionate, contrasting with his previous skepticism and brief answers. When using scales to measure his confidence about the change (how confident he was about success), his confidence was relatively low and relied on the hope that he could “push the boundaries” that he had set (that is, the list of what he does not want). We agreed that the first goal of our cooperation would be to shift the boundaries and the possibility of extending them.

The previous examples show that forming a preferred future and/or goals is not always a straightforward process. Generally, we assume that when clients come with problems or complaints, they/have a certain (albeit not clear) idea of what they want instead [58,63]. Although sometimes they can describe this idea in detail during the first meeting, in other cases negotiating the goals and direction of cooperation with the client requires more time (see Mr. B’s case), or the description of a preferred future mingles with the description of complaints, and sometimes it is demanding to make the client speak about their dreams (see Mrs. A’s case).

### 4.2. Resources

This area focuses on where clients currently are and on their past successes. The process of determining the current situation in the solution-focused approach is fundamentally different from the problem-oriented processes of “diagnosing” or “assessment” [27]. The main difference is focusing on resources instead of weaknesses and the sensitive work with language in conversation [33,39]. Focusing on resources is not about ignoring the limitations and shortcomings of the client but instead about consistently mapping what is available and possible instead of mapping what is lacking [43,58]. To do this, the worker focuses on the progress made toward the goals and what contributed to this (resources). 

#### 4.2.1. Case Example 1: Mrs. A. (Continuation)

I talked with the client about things that have already been carried out to achieve her dream. I was surprised to see how many steps the client had already taken, and I was curious about the details. The client described how she tried to improve her English (e.g., by listening to the Internet and reading English books). She also mentioned attending a recertification course (in the field of accountancy) and a computer skills course to refresh her knowledge. She consequently sent out job applications and paid attention to her CV to avoid the impression of being “overqualified” for certain positions. We talked about other things that she had managed or that she had mastered. We discussed her responsibility, willingness to learn new things, loyalty, sophisticated manners, and many other traits. The fact that she was asked further questions about specific cases of skills proved to be crucial. Telling these stories changed the atmosphere of the consultation; the client began, as she said, “being more aware of what she is good at”. It was not something new that she would not have known before, but now she can benefit from having very clear and lively ideas instead of abstract ideas. For example, we did not speak about the quality of her “reliability” (in the client’s terms) on some abstract label but rather about concrete manifestations of this “reliability” in the context of her everyday life situations.

We also talked about the people who support her—she mentioned her son and one close friend with whom she can discuss her concerns. An interesting moment occurred during the second session when she came up with the following summarization:

*“Maybe I’m trying to get a job that is not up to the standard I require, perhaps I do not trust myself enough to try something proper... now somehow I feel that I can dare to reach higher...”*

We spent the rest of the consultation discussing the reasons that brought her desire to reach higher ground, and it was all based on our prior consultation about the resources available.

#### 4.2.2. Case Example 2: Mr. B. (Continuation)

I told the client to focus on the situations when he overcame something he thought was impossible (“limit”). He remembered some situations during his studies and working at the post office. He realized that when the chips were down, he could excel, and he also had the support of his parents, with whom he lives in his family home. He also revived some contacts from the past (classmates and colleagues), and he created his own blog focusing on biology, as he is good at working with PCs. Although it was difficult to obtain some specific resources, the client admitted to having more confidence and determination.

These cases may seem at this point somehow abridged as if they were only lists of resources. However, it is important to describe these resources in the specific situations of the clients’ lives and especially in connection with their goals or dreams [58,64]. Compliments—one of the key techniques of the solution-focused approach—is not an attempt to be “nice” to clients but a deliberate and systematic promotion of eliciting clients’ resources in a conversation [63].

### 4.3. Action

After mapping where the client wants to go and how far they have already got (including what helped them get there), we can move on to the “action” frame. The path to the goal then begins with the first small step [64] or another version of the “experiment” between sessions, such as observing progress, pretending a small change happens, etc. [57,58,63]. 

#### 4.3.1. Case Example 1: Mrs. A. (Continuation)

We tried to describe to the client the next step toward her goal. The client was initially inclined to try intensive training in English and made a plan to work on her English with listening exercises three times a week. At the following meeting, she reported that she had not managed to improve, but she had been able to continue completing the listening activity regularly. We talked about what helped her to continue listening once per week. At the end of the second meeting, we agreed on another small step: the client decided that she would try to send out her job applications to three workplaces that she liked but had not dared to apply to before. Having completed this, she was invited for an interview by two of them. We discussed it in another session, and the client thought these job interviews could be understood as an experiment into whether she can “stand up with honor” and “sell what she can do”. Although she did not gain the job position in either case, her participation in the interviews confirmed her ability to partake in an interview and respond in a refined manner. Paradoxically, the failure did not decrease her self-confidence but helped her raise it. Perhaps this was also caused by the fact that, in one case, she had been the second most successful applicant. Moreover, she received very positive feedback in both cases. It helped her worry less about sending applications to a larger number of workplaces. Now she could dare to compete with others. A few weeks later, she won an open competition and obtained a job. It is a highly technical economic part-time job that the client could arrange with the employer. The employer originally sought a full-time employee, but because Mrs. A was the best applicant, he finally accepted her request to work only part-time.

#### 4.3.2. Case Example 2: Mr. B. (Continuation)

The first small step which we agreed on with the client was that he would send an application for such job positions, which “pushes the boundaries” that he had previously set. The client accepted it, as there was—in his words—“nothing to lose”. Gradually, he began to respond to job offers no matter if they required knowledge of English, and they were outside the city where he lived. In each session, we investigated his resources (what helped him to do it). Although he did not succeed at obtaining a job, he decided to improve his ability to speak English as he was responding to offers that counted on this ability. During the consultation, he came up with the idea to run his blog in English and practice English. This work was very entertaining for him. We also considered the possibility of addressing the companies that currently do not seek employees and the possibility of continuing the “revival of old contacts”. He was contacted by his former classmate, who was in touch with a research team of biologists in New Zealand. The client started considering going abroad and working as an assistant on the team for a limited period. It was exactly the branch that he had studied and enjoyed, and thanks to that, he might gain some interesting experience useful for future job applications in his home country.

On the other hand, this option significantly pushed almost all the “boundaries” which had originally been set. I appreciated this fact and talked with the client about the challenge he is about to face when “pushing these boundaries”, as well as the matter of confidence (where he gains his confidence from and how much confidence he has about taking such a step). Finally, with the help of a former classmate, he contacted the team of biologists, and by the time we ended our cooperation, he had already taken very specific steps to be able to fly to New Zealand.

## 5. Discussion

We have tried to introduce solution-focused coaching as a potentially promising way of working with unemployed clients in the context of social work counseling to enhance their well-being. The potential for this type of work has been documented by two brief case examples. 

A decline in self-esteem may affect in a particular way an individual who has shown identity creativity in the past (see case example 1 previous to maternity leave), but this is made more difficult by the limitation of one of the resources (available time, which is limited due to childcare in case example 1). Even though the individual’s other resources are not limited compared to the past, lowered self-esteem leads to orientation towards jobs below the level of one’s own qualifications. The fact that the key to the solution was reorienting the individual towards her own resources instead of limitations finally enabled, in case example 1, the client to obtain a part-time position that was originally planned by the employer as full-time. Ryff’s psychological well-being categories [19] of self-acceptance, environmental mastery, and personal growth are visible here. 

However, the threat to self-esteem also applies to an individual who does not care much about identity creativity, as was initially the case with client B (case example 2). He initially does not want to push his “boundaries”, he wants something staid where he was simply doing his job, and is constrained by a series of “boundaries” that seem insurmountable and, thus, significantly narrow his idea of his employability. In order to overcome the current tendency towards an “exit” type of reaction [14], the work of the coach also lies in fostering that identity creativity. Thus, client B is eventually able to imagine himself in a job position that requires aspects, initially rejected, and “constructing” resources in the coaching process.

The presented case examples show the importance of defining very small steps. When it comes to small steps, there is a relatively high chance of their accomplishment, which, together with the awareness of available resources, reinforces the client’s motivation [60,64]. Apart from that, the client gained new experience by accomplishing the goals that he had set for himself. The individual steps towards the goal often do not offer a straightforward path. Some steps, even though defined as small ones and supposedly feasible, fail to be realized. Unrealized steps do not necessarily mean the renunciation of the entire target, and the clients themselves sometimes find other ways to move forward. In the solution-focused approach, great emphasis is placed on the careful monitoring of changes and defining immediate small steps at the expense of the longer-term steps (e.g., the whole distance to the goal).

Looking back to Seligman’s five elements of psychological well-being [1], we can link the work with clients to all areas:

1. Positive emotions—there is a variety of positive emotions with a significant impact on broadening possibilities and building new resources [87]; positive emotions are present mainly during conversations about dreams/goals (e.g., hope, interest, inspiration, and joy) and about resources (e.g., gratitude, inspiration, pride, and hope) [88,89]. Emotional change is often visible (see the shining eyes of the client in the case of example 1); sometimes, it is more subtle but manifests itself by enhancing new possibilities and resources (see case example 2).
2. Engagement (flow)—engagement took place in sessions when clients were inspired and immersed themselves in some inspiring topic (such as long descriptions of dreams or successes). Even more, engagement was experienced by the clients in their life situation when they focused their attention differently on changes they made or their successes (see pushing boundaries in case example 2 or new job interviews in the case example 1).
3. Relationships—positive changes in relationships are often the results of asking for support (see “revival of old contacts” in example 2 or the change in the relationship of the client in case example 1 with her son and friend). Some studies have shown that a shared goal promotes cooperation even in conflicting groups [90], so the healing potential of goals and support in relationships is reasonably expected [91].
4. Meaning—meaning of life is strongly connected to well-being [19] and is very helpful in dealing with adversity and setbacks [85,92]. For both clients mentioned in the case examples, work meant a lot. Both of them found valuable goals, which helped them commit to them and overcome difficulties.
5. Achievement—focusing on even small successes or signs of improvement enhances the feeling of achievement, which contributes to well-being per se [1,19] and strengthens self-efficacy as well as gratitude and meaning in life [82]. Focusing on small achievements plays a crucial role in both case examples presented here and probably served as a basis for future major achievements, such as a new job in case example 1 and moving to New Zealand in case example 2.

## 6. Conclusions

Although more research is needed to establish the effectiveness of solution-focused coaching with unemployed clients, we assume that solution-focused coaching promotes key areas of psychological well-being and can be effectively used in work with unemployed clients. Particularly the focus on negotiating direction (preferred future) and resources (first two key areas of the Reteaming model) is related to elements of psychological well-being. Those areas should be emphasized in any work with the unemployed aimed at enhancing their psychological well-being. While innovating the education of future social workers or life-long learning for graduated social workers, it is recommended to focus more on methods and techniques which help to address those areas. 
